# Effects of *Withania somnifera* (Ashwagandha) and *Terminalia arjuna* (Arjuna) on physical performance and cardiorespiratory endurance in healthy young adults

**DOI:** 10.4103/0974-7788.72485

**Published:** 2010

**Authors:** Jaspal Singh Sandhu, Biren Shah, Shweta Shenoy, Suresh Chauhan, G. S. Lavekar, M. M. Padhi

**Affiliations:** *Department of Sports Medicine and Physiotherapy, Guru Nanak Dev University, Amritsar, Punjab - 143 005, India*

**Keywords:** Absolute and relative power, balance, blood pressure, maximum oxygen consumption (VO2 max), *Terminalia arjuna*, velocity, *Withania somnifera*

## Abstract

**Introduction:**

Several medicinal plants have been described to be beneficial for cardiac ailments in Ayurveda like Ashwagandha and Arjuna. Ashwagandha-categorised as Rasayanas, and described to promote health and longevity and Arjuna primarily for heart ailments. coronary artery disease, heart failure, hypercholesterolemia, anginal pain and can be considered as a useful drug for coronary artery disease, hypertension and ischemic cardiomyopathy.

**Objective:**

There are no scientific clinical studies showing effect of both these drugs on exercise performance after regular administration when given as supplements The present study was therefore designed and performed to assess the effects of *Withania somnifera* (Ashwagandha) and Terminalia arjuna (Arjuna) individually and as a combination on maximum velocity, average absolute and relative Power, balance, maximum oxygen consumption (VO2 max) and blood pressure in humans.

**Materials and Methods:**

Forty normal healthy. Subjects (either sex, mean age 20.6 ± 2.5yrs and mean Body Mass Index 21.9 ± 2.2) were recruited after written informed consent was obtained. Institutional Ethics Committee permission was also obtained. Thirty participants were assigned to experimental group of which 10 received standardized root extracts of *Withania somnifera*, 10 received standardized bark extract of Terminalia arjuna and the rest of the 10 received standardized root extract of *Withania somnifera* in addition to bark extract of Terminalia arjuna both. Both the drugs were given in the form of capsules (dosage 500mg/day for both the drugs). Ten participants received placebo (capsules filled with flour). All the subjects continued the regimen for 8 weeks. All variables were assessed before and after the course of drug administration

**Observations:**

Our study showed that *Withania somnifera* increased velocity, power and VO2 max whereas Terminalia arjuna increased VO2 max and lowered resting systolic blood pressure. When given in combination, the improvement was seen in all parameters except balance and diastolic blood pressure.

**Conclusion:**

*Withania somnifera* may therefore be useful for generalized weakness and to improve speed and lower limb muscular strength and neuro-muscular co-ordination. Terminalia arjuna may prove useful to improve cardio-vascular endurance and lowering systolic blood pressure. Both drugs appear to be safe for young adults when given for mentioned dosage and duration.

## INTRODUCTION

There is renewed interest in traditional medicines because of a perception of lower incidence of side effects. The World Health Organization (WHO) estimates that 80 percent of the world’s population presently uses herbal medicines for some aspect of primary health care.[1] Several medicinal plants have been described to be beneficial for cardiac ailments in Ayurveda - the origin of Indian system of Medicine.[2]

*Withania somnifera* (WS), also known as Ashwagandha, Indian ginseng, or winter cherry, has been an important herb in the Ayurvedic and indigenous medical systems for over 3000 years. The roots of the plant are categorised as *Rasayanas*, and described to promote health and longevity by augmenting defenses against disease, arresting the ageing process, revitalizing the body in debilitated conditions and thus creating a sense of wellbeing.[3] *Withania somnifera* contains alkaloids (withanine, withasomnin) and steroidal lactones and glycosides also called as withanoloids and sitoindosides and the extract of *Withania somnifera* has analgesic, mildly sedative, anti-inflammatory and anabolic activities,[4] and it is useful in stress, strain, fatigue, pain, skin diseases, diabetes, gastrointestinal disease, rheumatoid arthritis, and epilepsy,[5] chronic fatigue syndrome[6] and even during pregnancy without any side effects.[7] It is also used as a general tonic, to increase energy and improve health and longevity.[4] *Withania somnifera* human studies suggest that, it may promote growth in children and improve hemoglobin level, red blood cell count, and physical performance in adults.[4] *Terminalia arjuna* is widely used in both Ayurvedic and Unani Systems of medicine, primarily for heart ailments. *Terminalia arjuna* Wight and Arn. is a deciduous and evergreen tree, standing 20–30m above ground level and belongs to the Combretaceae family.[8,9] It is described as an alexteric, stryptic, tonic, and anthelmintic agent and is also useful in treatment of fractures, ulcers, heart diseases, biliousness, urinary discharges, asthma, tumours, leucoderma, anaemia, excessive perspiration[8] etc and its bark is useful in the treatment of coronary artery disease, heart failure, hypercholesterolemia, anginal pain[10] and can be considered as a useful drug for coronary artery disease, hypertension and ischemic cardiomyopathy.[11–13] *Terminalia arjuna* has also cardioprotective property,[14] antiviral activity against HSV-2[14,15] and efficiency as potent antioxidant preventing LDL cholesterol oxidation.[16,17]

There are no scientific clinical studies showing effect of herbal drugs on exercise performance after regular administration when given as supplements. This study was conducted to explore the effects of these two plants on physical and cardiovascular performance in healthy young adults.

## MATERIALS AND METHODS

### Research design

The present study was designed to be a randomized controlled, parallel group, single blinded study.

#### Sample

Forty healthy individuals of either sex (22 males and 18 females), with a mean age of 20.6 ± 2.5 (aged between 18 to 25 years) years and BMI 21.9 ± 2.2 kg/m^2^ (ranged between 18 to 25) from the population of Guru Nanak Dev University campus volunteered for the study. The sample size was calculated by online ‘Java applets for power and sample size’ software,[18] keeping power of the study at 95%. The subjects were randomly assigned into four groups using the chit in a box method. Group I (n=10): *Withania somnifera* group, Group II (n=10): *Terminalia arjuna* Group, Group III (n=10): *Withania somnifera* and *Terminalia arjuna* group, Group IV (n=10): Placebo (control) group. Subjects were unaware of which group they were in and which drug they were to receive. It was thus a single blinded study, where all the subjects were completely unaware of drugs which they were going to consume.

#### Selection of subjects

College going young adults with age between 18 and 25 years were screened. To avoid confounding effects, we included only those individuals who were free from any lower limb injury within past six months, those whose BMI was between 18 and 25 and who had not participated in regular exercises in gym from past 6 months or more.

Individuals who were engaged in regular strenuous physical activity, suffering from chronic illness or had undergone major surgery recently, were suffering from any cardiovascular, musculoskeletal or neurological condition and people with history of alcohol abuse or were under medication of other drugs were excluded.

#### Variables for effect

The following variables were assessed before and after drug administration under supervision and while ensuring safety of the subjects:

1. Kinematic Measuring System (KMS) ^™^ was used to measure maximum velocity. This instrument contains four cameras and they were placed at specific distance and at regular intervals to measure velocity. The participants were asked to sprint and at each phase of camera, the velocity was noted. The maximum velocity was calculated as maximum distance travelled at any phase of camera per second.
2. The same instrument (KMS) was used to measure average absolute and average relative power of the lower limbs. During 10 vertical jumps both the values were derived from the contact mat (automatically calculated kinematic values) and the body mass was calculated by associated software. Absolute power (W) = body mass × gravity × jump height / (contact time/2); Relative power = power (W)/ body mass.
3. A 20-second wobble board test (Kinematic) was performed, and a software program was used to calculate a balance ratio (contact with floor to no contact time). A metal plate connected to the computer hardware was placed under the wobble board. When the perimeter of the wobble board made contact with the metal plate, the duration and frequency (during the 20-second test) of contact was recorded by the software. Subjects received an orientation session for the balance board on a separate day, as well as 1–2 practice attempts on the day of testing.
4. Computer controlled Vista Turbo Trainer^™^ machine was used for evaluating breath by breath gas exchange kinetics. Peak maximum oxygen consumption (ml/kg/min) was measured by using software ‘Turbofit’ version – 5.04.
5. Sphygmomanometer was used to measure systolic and diastolic blood pressure. Resting blood pressure was taken in consideration.
6. Weighing machine (auto-inc) and kinanthropometric rod were used to measure body mass (kg) and vertical height (meter) to calculate Body Mass Index (BMI).

#### Procedure

The study was approved by the Institutional Medical Ethics Committee of Guru Nanak Dev University, Amritsar. Prior to the start of data collection, participants were explained about the drugs and previous research supporting the effectiveness on physical performance and possible side effects due to overdose. Only then the subjects who volunteered to participate in the study were recruited. A written informed consent was taken from each participant prior to recruitment. Only those subjects whose BMI was less than 25[19,20] were recruited.

### Test drugs

*Withania somnifera* was used in the form of a standardized aqueous root extract and Teminalia *arjuna* was in the form of aqueous bark extracts. The drugs were obtained from Central Council for Research in Ayurveda and Siddha (CCRAS), Delhi, India.

Both the drugs were filled in 500mg gelatin capsules. They were stored in air tight containers and in room temperature below 30°C throughout the experiment.

Both drugs were given in the dose of 1 capsule/day orally for 8 weeks.

The compliance of the participant to study drug was ensured as the researcher personally administered the drug to the subjects over the period of 8 weeks.

All variables mentioned above were measured before and after 8 weeks of drug administration in Isotonic and VO_2_ max lab and KMS lab in Department of Sports Medicine and Physiotherapy, Guru Nanak Dev University, Amritsar

### Monitoring of subjects

All subjects were healthy college going young adults with moderately active life style. The subjects were instructed to follow the usual routine without any excess physical exertion or exercises throughout the duration of experiment. All the subjects consumed the same meals given in the hostel mess throughout the procedure and were requested to have meals within specified mess time, when the researcher was present and personally administered the drugs. Volunteers were asked to consume the drug 1 hour after the day meal to maintain uniformity of the drug administration of *Withania somnifera* and *Terminalia arjuna*. Though the subjects were informed about possible side effects of the drugs in high dosage, subjects were also asked to report immediately if they feel any side-effect of the drugs but none of them felt any kind of the side-effect.

### Statistical analysis

The data was analyzed for statistical significance by using the Statistical Package for Social Sciences (SPSS 17.0) software. The student‘t’ test and one way ANOVA were used to analyze the data for the level of significance. The related‘t’ test was used to find intragroup and ANOVA was used to find intergroup differences in pre and post protocol. For all analysis, the *P* value used for statistical significance was 0.05. All results are expressed as mean ± standard deviation.

## RESULTS

After 8 weeks treatment with *Withania somnifera*, maximum oxygen consumption increased significantly from 13.54±2.46 to 14.47±2.28 (*P*=0.005). Similarly, the maximum velocity increased from 5.37±0.75 to 5.53±0.70 (*P*=0.005), the average absolute power from 711.90±221.62 to 774.79±247.42 (*P*=0.002) and average relative power from 11.10±3.17 to 12.22±3.40 (*P*=0.007). However, there was no significant improvement in balance and blood pressure. Table 1 summarizes these results.

**Table 1:** Effects of Withania somnifera

Parameters	*Withania somnifera*	Mean ± SD	*P* value
Max velocity	Pre test	5.37±0.75	0.005
	Post test	5.53±0.70	
Avg absolute power	Pre test	711.90±221.62	0.002
	Post test	774.79±247.42	
Avg relative power	Pre test	11.10±3.17	0.007
	Post test	12.22±3.40	
Balance	Pre test	0.84±0.34	0.412
	Post test	0.93±0.33	
VO2 max	Pre test	13.54±2.46	0.000
	Post test	14.47±2.28	
Systolic blood pressure	Pre test	120.20±3.58	0.591
	Post test	119.80±3.19	
Diastolic blood pressure	Pre test	78.40±3.10	0.443
	Post test	78.80±2.70	

The volunteers receiving s treatment with *Terminalia arjuna* demonstrated significant increase in maximum oxygen consumption capacity from 14.34±2.94 to 15.04±2.76. The systolic blood pressure fell significantly from 123.00±2.87 to 117.80±1.48 mmHg. The average absolute power also increased significantly from 656.20±220.78 to 680.00±232.51 (*P*=0.024). None of the other parameters showed significant change. This data is summarized in Table 2.

**Table 2:** Effects of *Terminalia arjuna*

Parameters	*Terminalia arjuna*	Mean ± SD	*P* value
Max velocity	Pre test	5.19±0.80	0.180
	Post test	5.15±0.81	
Avg absolute power	Pre test	656.20±220.78	0.024
	Post test	680.00±232.51	
Avg relative power	Pre test	10.29±2.56	0.671
	Post test	10.34±2.59	
Balance	Pre test	0.83±0.33	0.82
	Post test	0.84±0.28	
VO2 max	Pre test	14.34±2.94	0.000
	Post test	15.04±2.76	
Systolic blood pressure	Pre test	123.00±2.87	0.000
	Post test	117.80±1.48	
Diastolic blood pressure	Pre test	78.80±2.35	1.000
	Post test	78.80±1.69	

Table 3 shows comparison of variables before and after drug administration in group III (Witahnia somnifera and *Terminalia arjuna*). A significant improvement was seen in average absolute power from 793.61±286.00 to 883.49±274.00 (*P*=0.000), average relative power from 11.10±3.78 to 12.22±3.69 (*P*=0.000), maximum oxygen consumption from 16.58±4.70 to 17.70±4.51 (*P*=0.000), maximum velocity from 5.12±0.86 to 5.21±0.89. The systolic blood pressure fell from 123.40±3.13 to 118.00±2.49 (*P*=0.000).

**Table 3:** *Withania somnifera* and *Terminalia arjuna*

Parameters	*Withania somnifera* + *Terminalia arjuna*	Mean ± SD	*P* value
Max velocity	Pre test	5.12±0.86	0.004
	Post test	5.21±0.89	
Avg absolute power	Pre test	793.61±286.00	0.000
	Post test	883.49±274.00	
Avg relative power	Pre test	11.10±3.78	0.000
	Post test	12.22±3.69	
Balance	Pre test	0.72±0.31	0.922
	Post test	0.72±0.28	
VO2 max	Pre test	16.58±4.70	0.000
	Post test	17.70±4.51	
Systolic blood pressure	Pre test	123.40±3.13	0.000
	Post test	118.00±2.49	
Diastolic blood pressure	Pre test	78.60±3.53	0.619
	Post test	78.20±1.48	

In comparison, 8 weeks of regular administration of placebo to the control group showed no significant changes in any of the variables [Table 4].

**Table 4:** Effects of placebo

Parameters	Placebo	Mean ± SD	*P* value
Max velocity	Pre test	5.30±0.70	0.462
	Post test	5.54±0.75	
Avg absolute power	Pre test	718.29±280.37	0.258
	Post test	726.82±279.96	
Avg relative power	Pre test	10.77±3.36	0.556
	Post test	10.84±3.16	
Balance	Pre test	0.92±0.35	0.974
	Post test	0.92±0.29	
VO2 Max	Pre test	16.02±2.91	0.825
	Post test	16.06±2.54	
Systolic blood pressure	Pre test	121.80±3.58	0.798
	Post test	121.60±1.84	
Diastolic blood pressure	Pre test	79.40±2.99	0.780
	Post test	79.60±2.07	

Table 5 shows intergroup comparison of maximum velocity, average absolute power, average relative power, maximum oxygen consumption, and systolic as well as diastolic blood pressure after 8 weeks of drug administration. A significant reduction in resting systolic blood pressure was seen in only group II when groups were compared with each other. ANOVA followed by Post Hoc Multiple Scheffe Range Test after completion of drug dosage showed that group II (*Terminalia arjuna*) significantly effective (F= 5.757, *P*= 0.003) in reducing systolic blood pressure [Table 5]. There is no statistically significant difference found in any other parameters when the all four groups were compared with each other.

**Table 5:** Intergroup comparison of all parameters (One way ANOVA)

		Sum of Squares	df	Mean Square	F	Sig. (p)
Max Velocity	Between groups	0.853	3	0.284	0.456	0.714
	Within groups	22.425	36	0.623		
	Total	23.278	39			
Avg Abs Power	Between groups	228120.9	3	76040.29	1.132	0.349
	Within groups	2418566	36	67182.39		
	Total	2646687	39			
Avg Rel Power	Between groups	44.083	3	14.694	1.403	0.258
	Within groups	377.145	36	10.476		
	Total	421.227	39			
VO2 max (ml/kg)	Between groups	60.229	3	20.076	2.025	0.128
	Within groups	356.909	36	9.914		
	Total	417.138	39			
Systolic blood pressure	Between groups	94.8	3	31.6	5.757	0.003
	Within groups	197.6	36	5.489		
	Total	292.4	39			
Dialostic blood pressure	Between groups	9.9	3	3.3	0.796	0.504
	Within groups	149.2	36	4.144		
	Total	159.1	39			

## DISCUSSION

The present study was aimed to assess the effects of *Withania somnifera* and *Terminalia arjuna* singly and in combination *Withania somnifera* and *Terminalia arjuna* on physical performance and endurance in healthy young adults after an eight week therapy. Maximum velocity, average power (absolute and relative) and balance were measured as physical performance parameters and maximum oxygen consumption and blood pressure as were measured as endurance parameters. Both, the maximum velocity and average power represent short term aerobic activity whereas VO_2_ max represents long term aerobic and cardiovascular endurance. Balance is an ability to maintain Centre of Gravity (COG) within the base of support with minimal postural sway. It requires integration of inputs from multiple senses.

Ayurveda is a rich heritage of herbal practices describing medicinal and nutritional uses of more than 600 plants in seventy books. Many plants have ergogenic effects, with no or very less side effects. Ginseng is known as an adaptogen, which means it increases resistance to physical, chemical, and biological stress and builds energy and general vitality.[21] *Withania somnifera* is considered to be the “Indian” ginseng.[22] We found that *Withania somnifera* improved the physical performance and strength parameters in our study after 8 weeks of regular consumption (500mg/day). Singh *et al*.[7] have described use of *Withania somnifera* in chronic fatigue syndrome. It helps in delaying onset of fatigue and thus increasing the time for exhaustion and maintaining the power for relatively longer period. In our study, the maximum velocity, average absolute and relative power increased by 2.9%, 8.8% and 10.1% respectively following drug administration compared to the placebo group. Arman *et al*.,[23] reported that *Withania somnifera* (improves endurance performance (time to exhaustion) at a moderate intensity of 65% VO_2_ max, in untrained healthy individuals. In the present study, we found that following 8 weeks of administration of *Withania somnifera* maximum oxygen consumption capacity increased by 6.8% at moderate intensity but no significant change was seen in balance and resting blood pressure.

*Terminalia arjuna* is a cardio protective drug and is used in ayurveda since centuries for its cardiotonic properties. The present study shows that there is significant improvement in average absolute power of lower limbs by 3.6%. Bharani *et al*, observed significant improvement in the duration of treadmill exercise in stable angina patients who received *Terminalia arjuna* when given 500 mg/day for one week.[24] In our study, we found an increase in maximum oxygen consumption capacity by 4.9% after treatment. In animal studies, Ghoshal *et al*.[25] reported an increased heart rate and force of contraction in cardiac muscles in isolated rats. Shrivastava *et al*. found a dose dependant fall in blood pressure in rats when *Terminalia arjuna* bark was given in aqueous form, intravenously.[26] According to Colabawala (1951), the drug is known to have no significant effect on heart rate, blood pressure and cardiac output in healthy volunteers but causes an increase in cardiac output and blood pressure and a decrease in heart rate in patients with a failing heart.[27] Contradicting this statement, in our study we found that, there is significant decrease in systolic blood pressure by 4.2% when compared with placebo group [group IV] but no significant improvement was seen in diastolic blood pressure in healthy young adult volunteers following 8 weeks of *Terminalia arjuna* bark extract consumption.

When *Withania somnifera*and and *Terminalia arjuna* were given in combination in group III, all parameters showed significant improvement except balance and diastolic blood pressure. The maximum velocity, average absolute power, average relative power, VO_2_ max and systolic blood pressure improved by 1.8%, 11.3%, 10.1%, 6.8% and 4.4% respectively in its group when compared with placebo group [group IV].

When results between groups were compared the group which was given both *Terminalia arjuna* and *Withania somnifera* (group III) was the most effective in reducing systolic blood pressure (4.37%), which is highest significant reduction in systolic blood pressure between groups followed by group II (4.22%) that consumed only *Terminalia arjuna*. There is no significant difference were seen for any other parameters Without training or excessive physical exertion, *Terminalia arjuna* was found to be effective in reducing resting systolic blood pressure in healthy young adults.

The maximum velocity was found to be improved the most in the *Withania somnifera* treated group followed by the group that received both *Withania somnifera* and *Terminalia arjuna*. Average absolute power was found to be improved most in the *Withania somnifera* and *Terminalia arjuna* group, followed by *Withania somnifera* group and *Terminalia arjuna* group respectively. *Withania somnifera* and *Terminalia arjuna* were equally effective in improving relative power of the lower limbs. The maximum oxygen consumption capacity was effectively increased in those subjects, who were given *Withania somnifera* and *Terminalia arjuna* in combination followed by those who were given just *Terminalia arjuna*.

The present study was limited to an 8 week period on healthy young adults. The future research should focus on longer treatment duration, dose finding as well as gender specific effects of the drugs. Further studies are also required to assess whether the drugs can improve other physical parameters and to see the effectiveness in elite sports persons so that in future these drugs can be given as ergogenic elements.

*Withania somnifera* may therefore be useful for generalized weakness and to improve speed and lower limb muscular strength and neuro-muscular co-ordination. *Terminalia arjuna* may prove useful to improve cardio-vascular endurance and lowering systolic blood pressure. Both drugs appear to be safe for young adults when given for mentioned dosage and duration.
